# Associations of the Life’s Essential 8 with Parkinson’s disease: a population-based study

**DOI:** 10.3389/fnagi.2025.1510411

**Published:** 2025-02-18

**Authors:** Chenguang Zhou, Oumei Cheng

**Affiliations:** Department of Neurology, The First Affiliated Hospital of Chongqing Medical University, Chongqing, China

**Keywords:** Life’s Essential 8, Parkinson’s disease, NHANES, cardiovascular health, weighted quantile sum (WQS) regression

## Abstract

**Background:**

Parkinson’s disease (PD) is a progressive neurodegenerative disorder with increasing global prevalence. This study investigated the association between the American Heart Association’s Life’s Essential 8 (LE8) and PD prevalence using a large, nationally representative database.

**Methods:**

We analyzed data from 18,277 participants aged 40 years and older from the National Health and Nutrition Examination Survey (NHANES) 2005–2018. LE8 scores were calculated based on diet, physical activity, nicotine exposure, sleep, body mass index, blood lipids, blood glucose, and blood pressure. PD cases were identified through self-reported anti-PD medication use. Multivariate logistic regression models were employed to examine the association between LE8 and PD prevalence, adjusting for various demographic and clinical factors. In addition, we performed restricted cubic splines (RCS), subgroup analyses, and weighted quantile sum (WQS) regression to verify the robustness of the study results.

**Results:**

The prevalence of PD was 1.3% in the study population. After full adjustment, individuals with moderate (50–79) and high (80–100) LE8 scores showed lower odds of PD compared to those with low (0–49) scores (OR 0.53, 95% CI 0.29–0.97 and OR 0.43, 95% CI 0.17–1.04, respectively; *p* for trend <0.05). A dose-response relationship was observed between LE8 scores and PD prevalence. WQS regression identified dietary factors and glycemic health as the main contributors to the inverse association between LE8 and PD.

**Conclusion:**

Our findings suggest a significant inverse association between Life’s Essential 8 (LE8) and PD prevalence, with dietary factors and glycemic health emerging as the most influential components.

## 1 Introduction

Parkinson’s disease (PD) is a progressive neurodegenerative disorder affecting millions worldwide. Over the past generation, the global disease burden of PD has more than doubled, with an estimated 6.1 million cases in 2016 (GBD 2016 Parkinson’s Disease Collaborators, 2018). This upward trend is projected to continue, with predictions suggesting PD cases may reach 12 million by 2040 (Dorsey et al., 2018). The substantial economic and social costs associated with PD, coupled with its impact on patient’s quality of life, underscore the urgent need to identify modifiable risk factors for prevention and early intervention. Research conducted by Paul et al. (2019) has demonstrated the potential influence of lifestyle factors on PD risk. Their findings indicate that coffee, caffeinated tea, moderate alcohol consumption, and physical exercise may have protective effects against PD, while smoking and excessive alcohol use are associated with increased risk. Furthermore, several studies have suggested that PD may be linked to various risk factors, including body mass index (Hu et al., 2006), diet (Knight et al., 2022), nicotine exposure (Quik et al., 2008), alcohol consumption (Bettiol et al., 2015), regular exercise (Bhalsing et al., 2018), sleep disorders (Dodet et al., 2024), diabetes (Athauda et al., 2022), hypertension (Shi et al., 2024), and dyslipidemia (Lee et al., 2023). Given these associations, it is crucial to explore the relationship between comprehensive health indicators and the development and progression of PD.

Mounting evidence suggests a complex interplay between cardiovascular health and neurodegenerative diseases, including Parkinson’s disease (PD). Various cardiovascular risk factors, such as hypertension (Shi et al., 2024), diabetes (Athauda et al., 2022), and obesity (Hu et al., 2006), have been demonstrated to be associated with PD risk. The American Heart Association’s Life’s Simple 7 (LS7), a measure of ideal cardiovascular health, has been shown to correlate with reduced risk of cognitive decline and dementia (Samieri et al., 2018). Recently, LS7 was updated to Life’s Essential 8 (LE8), incorporating sleep as a crucial component of cardiovascular and brain health (Lloyd-Jones et al., 2022). LE8 is a multidimensional tool designed to assess overall health by evaluating diet, physical activity, nicotine exposure, sleep, BMI, blood lipids, blood glucose, and blood pressure. Although originally developed for cardiovascular health, these factors also exhibit potential relevance to PD. This study utilized LE8 as a tool to assess factors associated with Parkinson’s disease, based on the extensive overlap between these factors in cardiovascular health and neurodegenerative diseases. Previous research has shown that lifestyle and metabolic health factors, such as diet (Knight et al., 2022), physical activity (Bhalsing et al., 2018), sleep (Dodet et al., 2024), obesity (Hu et al., 2006), abnormal blood glucose levels (Athauda et al., 2022), and changes in lipid profiles (Lee et al., 2023), are closely linked to the risk and progression of PD. Given the potential shared pathophysiological mechanisms between cardiovascular diseases and PD, investigating the relationship between LE8 and PD risk presents a promising avenue for identifying novel preventive strategies and understanding the broader impact of cardiovascular health on neurodegenerative processes. This approach may provide valuable insights into the intricate connections between cardiovascular well-being and neurological health, potentially leading to more comprehensive and effective interventions for PD.

To address this knowledge gap, data from the National Health and Nutrition Examination Survey (NHANES) spanning 2005–2018 were utilized. NHANES provides a unique opportunity to examine the relationship between Life’s Essential 8 (LE8) and Parkinson’s disease (PD) in a large, nationally representative sample of the U.S. population. This dataset allows for a comprehensive assessment of cardiovascular health metrics, including the newly added sleep component, as well as PD status and relevant covariates. The present study aims to elucidate potential associations between LE8 scores and PD prevalence by leveraging this extensive database. This investigation contributes to the growing body of evidence regarding modifiable risk factors for PD and may inform future preventive strategies.

## 2 Methods

### 2.1 Study participants

**Figure 1:**
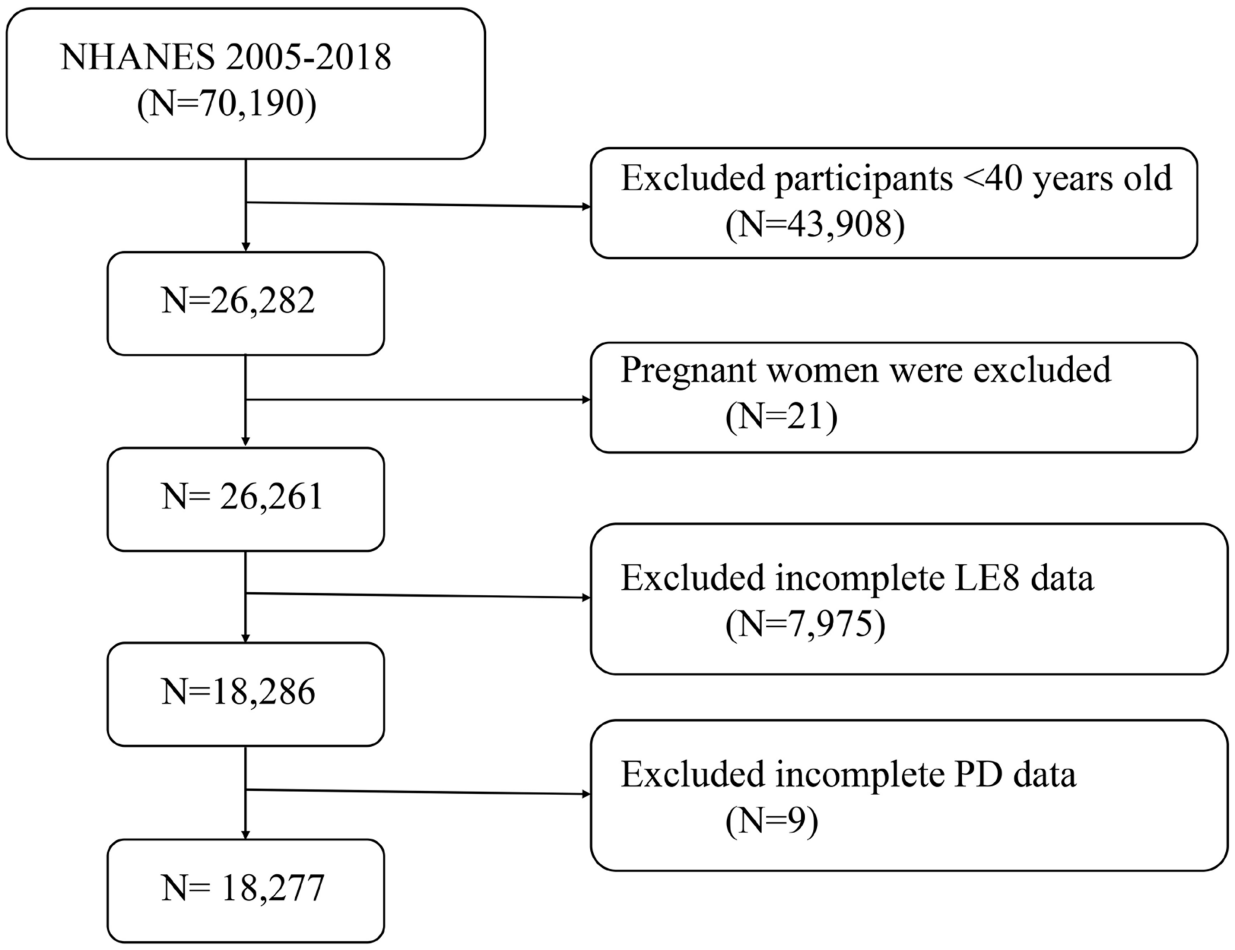
A flow diagram of eligible participant selection in the National Health and Nutrition Examination Survey. LE8, Life’s Essential 8; PD, Parkinson’s disease.

The National Health and Nutrition Examination Survey (NHANES) is a nationally representative cross-sectional survey conducted in the United States through household interviews and mobile examination centers. It evaluates the health and nutritional status of the American population. This study utilized data from seven NHANES cycles between 2005 and 2018, involving 70,190 participants. After excluding individuals younger than 40 years (*n* = 43,908), pregnant women (*n* = 21), and those with missing or incomplete data on LE8 or PD (*n* = 7,984), the final analysis included 18,277 participants. Figure 1 illustrates the flowchart of the selection process. NHANES is approved by the Research Ethics Review Board of the National Center for Health Statistics, with informed consent obtained from all participants. The data used in this study are de-identified and publicly available.^1^

### 2.2 Definition of Life’s Essential 8

LE8 is a refined and comprehensive framework introduced by the American Heart Association (AHA) to assess and enhance cardiovascular health (CVH). LE8 includes eight key components: four health behaviors (diet, physical activity, nicotine exposure, and sleep duration) and four health metrics [body mass index (BMI), blood lipids, blood glucose, and blood pressure] (Xiao et al., 2024). These components collectively contribute to an individual’s cardiovascular health status, which is crucial for preventing cardiovascular diseases (CVD) and improving overall life expectancy. Calculating a LE8 score involves quantifying each component based on established clinical guidelines and scoring systems. Diet is assessed using a dietary assessment tool, such as the Healthy Eating Index (HEI) 2015 (Supplementary Table S2), which evaluates adherence to dietary recommendations. Physical activity is measured by the total minutes of moderate or vigorous exercise per week, with a target of at least 150 or 75 min of moderate intensity. Nicotine exposure is determined by smoking status and exposure to secondhand smoke, with non-smokers and those avoiding secondhand smoke scoring higher. Sleep duration is evaluated based on the average hours of sleep per night, with 7–9 h considered optimal. The four health metrics are similarly quantified. BMI is calculated by dividing a person’s weight in kilograms by the square of their height in meters, with a normal range of 18.5–24.9 kg/m^2^ being ideal. Blood lipids are measured using the levels of non-high-density lipoprotein cholesterol (non-HDL cholesterol), aiming for levels below 130 mg/dL. Blood glucose levels are assessed by fasting blood glucose or HbA1c levels, with targets below 100 mg/dL and less than 5.7%, respectively. Finally, blood pressure is measured, with an optimal reading being less than 120/80 mm Hg. AHA has developed a new scoring system for each measure, with a range of 0 to 100 points (Liu et al., 2024). This allows for the creation of a new composite CVH score, which likewise has a range of 0 to 100 points. A higher score indicates a better state of health. The AHA recommendation said that an LE8 score of 80 to 100 points was regarded as high CVH, 50 to 79 points was considered moderate CVH, and 0 to 49 points was considered low CVH (Yang et al., 2024). Supplementary Table S1 has comprehensive explanations of how to use NHANES data to calculate scores for each LE8 indicator.

### 2.3 Diagnosis of Parkinson’s disease

The main outcome of this investigation was PD. According to previous studies (Huang et al., 2024; Tu et al., 2024), based on self-reported anti-PD medication use, PD cases have been found in the NHANES database. The following list of PD medications, such as benztropine, methyldopa, carbidopa, levodopa, entacapone, amantadine, and ropinirole, is used to diagnose PD.

### 2.4 Covariables

Based on previous research (Zhang et al., 2024; Liu et al., 2023), the covariates included in the study are age, sex, race, marital status, education level, family poverty-income ratio (PIR), smoking status, alcohol consumption, hypertension, diabetes, and hypercholesterolemia. Detailed descriptions of these covariates are provided in Supplementary Table S3.

### 2.5 Statistical analyses

Statistical analyses were conducted using R software (version 4.3.1). Sampling weights were applied in all statistical analyses to guarantee the national representativeness of the calculated data. “WTMEC2YR” was used as the weighting variable in our study, and the new weights (2005–2018) were computed as 1/7 × WTMEC2YR. Data were weighted as specified, with continuous variables presented as mean ± standard deviation, and *p*-values computed using weighted Students *t*-test. For categorical variables, percentages (weighted *N*, %) and *p*-values were calculated using weighted chi-square tests (Gong et al., 2024). The association between LE8 and PD was examined using multivariate logistic regression models. Three models were constructed: Model 1: no covariate adjustment; Model 2: adjusted for age, sex, education level, marital status, PIR, and race; Model 3: adjusted for age, sex, education level, marital status, PIR, race, smoking, alcohol consumption, hypertension, diabetes, and hypercholesterolemia. Covariates were selected based on their established associations with both LE8 and PD, as supported by prior literature. In Model 2, demographic and socioeconomic factors (age, sex, education level, marital status, PIR, and race) were included to account for their potential confounding effects. Model 3 further adjusted for lifestyle behaviors and chronic conditions (smoking, alcohol consumption, hypertension, diabetes, and hypercholesterolemia) that could act as intermediates or confounders in the association between LE8 and PD. Smooth curve fitting was used to further explore potential non-linear relationships between LE8 and PD. Subgroup analyses were performed to assess the association between LE8 and PD across different strata. Odds ratios (OR) were calculated as per 10 scores increase in LE8. Analyses were adjusted for age, sex, education level, marital status, PIR, race, smoking, alcohol consumption, hypertension, diabetes, and hypercholesterolemia. Additionally, we applied the WQS method to explore the overall effect of individual LE8 on PD. Nutrients with a WQS weighting (ranging from 0 to 1 and summing to 1) higher than 0.125 (the average of 8 LE8 metric) were identified as major contributors (Huang et al., 2024). Weighted quantile sum (WQS) regression assigns weights to the eight components of LE8, using a quantile regression optimization algorithm to determine their relative contributions to the risk of PD. The weights are normalized to sum to 1, with a threshold of 0.125 (1/8) serving as a reference value for comparison. WQS regression effectively handles multicollinearity and evaluates synergistic effects among variables, making it well-suited for high-dimensional analyses. To verify the robustness of the results, we excluded participants with a body mass index <18.5 and a history of cardiovascular disease and reanalyzed them using data from the NHANES 2007–2018 cycle. A *p*-value <0.05 was considered statistically significant.

## 3 Results

### 3.1 Characteristics of the participants

Our analysis included 18,277 participants, representing approximately 110,202,873 individuals. Table 1 outlines the general characteristics of the weighted study population, comparing those with and without PD. The prevalence of PD was 1.3% (equivalent to 1,463,538 individuals) with a mean (SD) LE8 score of 65.83 (14.22). Notably, most of the LE8 and its subgroups had lower scores in the PD group compared to the non-PD group. Significant differences were observed across various demographic and medical factors, such as gender, race, and hypertension (*p* < 0.05).

**Table 1:** Baseline characteristics of all participants were stratified by PD, weighted.

Characteristic	Overall, *N* = 110,202,873 (100%)	Non-PD, *N* = 108,739,335 (98.7%)	PD, *N* = 1,463,538 (1.3%)	*p*-value
No. of participants in the sample	18,277	18,034	243	-
Age (%)				0.060
40–60	68,072,992 (62%)	67,289,480 (62%)	783,512 (54%)	
>60	42,129,880 (38%)	41,449,855 (38%)	680,025 (46%)	
Gender (%)				**0.003**
Male	51,622,456 (47%)	51,112,397 (47%)	510,059 (35%)	
Female	58,580,417 (53%)	57,626,938 (53%)	953,479 (65%)	
Race (%)				**<0.001**
Non-Hispanic White	82,901,819 (75%)	81,625,502 (75%)	1,276,317 (87%)	
Non-Hispanic Black	10,241,800 (9.3%)	10,149,839 (9.3%)	91,960 (6.3%)	
Other	10,827,168 (9.8%)	10,771,502 (9.9%)	55,665 (3.8%)	
Mexican American	6,232,086 (5.7%)	6,192,491 (5.7%)	39,596 (2.7%)	
Married/live with partner (%)				0.127
No	34,292,121 (31%)	33,747,557 (31%)	544,564 (37%)	
Yes	75,910,752 (69%)	74,991,778 (69%)	918,974 (63%)	
Education level (%)				0.172
Below high school	16,385,052 (15%)	16,109,960 (15%)	275,091 (19%)	
High School or above	93,817,821 (85%)	92,629,374 (85%)	1,188,447 (81%)	
PIR (%)				0.110
Not poor	86,617,980 (84%)	85,561,170 (84%)	1,056,810 (79%)	
Poor	16,266,336 (16%)	15,991,405 (16%)	274,931 (21%)	
Smoking (%)				0.115
Never	57,208,305 (52%)	56,445,236 (52%)	763,068 (52%)	
Former	34,282,748 (31%)	33,910,372 (31%)	372,377 (25%)	
Current	18,711,820 (17%)	18,383,727 (17%)	328,093 (22%)	
Drinking (%)				0.159
Former	17,486,284 (17%)	17,150,167 (17%)	336,118 (24%)	
Heavy	14,639,073 (14%)	14,476,730 (14%)	162,343 (12%)	
Mild	43,441,407 (42%)	42,915,657 (42%)	525,750 (38%)	
Moderate	17,050,059 (16%)	16,855,796 (17%)	194,262 (14%)	
Never	10,917,270 (11%)	10,754,309 (11%)	162,961 (12%)	
Hypertension (%)				**0.014**
No	55,024,656 (50%)	54,448,781 (50%)	575,875 (39%)	
Yes	55,178,217 (50%)	54,290,554 (50%)	887,663 (61%)	
Diabetes (%)				0.197
No	91,006,506 (83%)	89,844,170 (83%)	1,162,336 (79%)	
Yes	19,196,366 (17%)	18,895,165 (17%)	301,202 (21%)	
High cholesterol (%)				0.306
No	54,239,639 (52%)	53,555,650 (52%)	683,989 (48%)	
Yes	49,860,036 (48%)	49,109,904 (48%)	750,132 (52%)	
Mean LE8 score [mean (SD)]	65.83 (14.22)	65.89 (14.21)	61.31 (14.71)	**<0.001**
Mean HEI-2015 diet score [mean (SD)]	42.51 (31.43)	42.58 (31.44)	37.23 (30.00)	0.056
Mean physical activity score [mean (SD)]	67.80 (42.79)	67.91 (42.73)	59.27 (45.94)	**0.039**
Mean tobacco exposure score [mean (SD)]	72.71 (36.74)	72.77 (36.68)	67.63 (40.55)	0.386
Mean sleep health score [mean (SD)]	83.33 (24.35)	83.41 (24.28)	77.44 (28.40)	**0.006**
Mean body mass index score [mean (SD)]	58.62 (33.06)	58.66 (33.01)	55.50 (36.80)	0.390
Mean blood lipid score [mean (SD)]	59.37 (29.43)	59.35 (29.42)	61.47 (29.64)	0.456
Mean blood glucose score [mean (SD)]	80.98 (26.42)	81.03 (26.39)	77.78 (27.87)	0.087
Mean blood pressure score [mean (SD)]	61.32 (31.56)	61.42 (31.55)	54.14 (32.12)	**0.010**
LE8 (%)				**<0.001**
Low (0–49)	14,664,730 (13%)	14,308,056 (13%)	356,674 (24%)	
Moderate (50–79)	76,390,803 (69%)	75,463,674 (69%)	927,128 (63%)	
High (80–100)	19,147,340 (17%)	18,967,604 (17%)	179,736 (12%)	

LE8, Life’s Essential 8; PD, Parkinson’s disease; PIR, ratio of family income to poverty; HEI-2015, Healthy Eating Index-2015. Mean (SD) for continuous variables: the *p*-value was calculated by the weighted Students *t*-test. Percentages (weighted *N*, %) for categorical variables: the *p*-value was calculated by the weighted chi-square test.

### 3.2 Association between LE8 and PD

Table 2 illustrates the relationships between LE8 and PD. The multivariate adjusted analyses showed that moderate (50–79) and high (80–100) were associated with a lower prevalence of PD compared to low (0–49), with odds ratios (ORs) and 95% confidence intervals (CIs) of 0.53 (0.29, 0.97) and 0.43 (0.17, 1.04), respectively (*p* for trend <0.05). Similar results were observed when LE8 was analyzed as a continuous variable. Additionally, in the fully adjusted model, all LE8 subgroups except the lipid score remained negatively associated with PD. Sensitivity analyses that excluded participants with a body mass index <18.5 and a history of cardiovascular disease showed robust results (Supplementary Table S5). In addition, the results of sensitivity analyses using fasting weights remained consistent (Supplementary Table S6).

**Table 2:** Adjusted odds ratios for CVH (LE8) and components to PD, weighted.

Characteristic	Model 1 [ORs (95% CI)]	*p*-value	Model 2 [ORs (95% CI)]	*p*-value	Model 3 [ORs (95% CI)]	*p*-value
Continuous (per 10 scores)	0.80 (0.72, 0.90)	<0.001	0.81 (0.71, 0.92)	0.001	0.81 (0.66, 0.99)	0.047
Low (0–49)	1 (Ref.)		1 (Ref.)		1 (Ref.)	
Moderate (50–79)	0.49 (0.33, 0.74)	<0.001	0.53 (0.34, 0.82)	0.005	0.53 (0.29, 0.97)	0.040
High (80–100)	0.38 (0.21, 0.70)	0.002	0.39 (0.20, 0.75)	0.005	0.43 (0.17, 1.04)	0.060
*p* for trend	<0.001		0.002		0.042	
Components (per 10 scores)						
HEI-2015 diet score	0.95 (0.90, 0.99)	0.048	0.93 (0.87, 0.98)	0.014	0.93 (0.87, 0.99)	0.027
Physical activity score	0.96 (0.92, 0.99)	0.020	0.97 (0.93, 1.01)	0.200	0.97 (0.93, 1.02)	0.300
Tobacco exposure score	0.97 (0.92, 1.01)	0.110	0.97 (0.92, 1.02)	0.200	0.81 (0.66, 0.99)	0.038
Sleep health score	0.92 (0.87, 0.97)	0.002	0.91 (0.85, 0.97)	0.004	0.91 (0.85, 0.99)	0.020
Body mass index score	0.97 (0.92, 1.02)	0.300	0.97 (0.92, 1.03)	0.200	0.98 (0.93, 1.04)	0.500
Blood lipid score	1.03 (0.96, 1.09)	0.400	1.04 (0.97, 1.11)	0.300	1.04 (0.96, 1.13)	0.300
Blood glucose score	0.96 (0.91, 1.00)	0.069	0.96 (0.91, 1.02)	0.200	0.96 (0.89, 1.05)	0.400
Blood pressure score	0.93 (0.89, 0.98)	0.006	0.94 (0.89, 0.99)	0.028	0.96 (0.89, 1.04)	0.300

CVH, cardiovascular health; LE8, Life’s Essential 8; PD, Parkinson’s disease; PIR, ratio of family income to poverty; HEI-2015, Healthy Eating Index-2015. Model 1: no covariates were adjusted. Model 2: age, gender, education level, marital, PIR, and race were adjusted. Model 3: age, gender, education level, marital, PIR, race, smoking, drinking, hypertension, diabetes, and high cholesterol were adjusted. A higher CVH score indicates better cardiovascular health.

**Figure 2:**
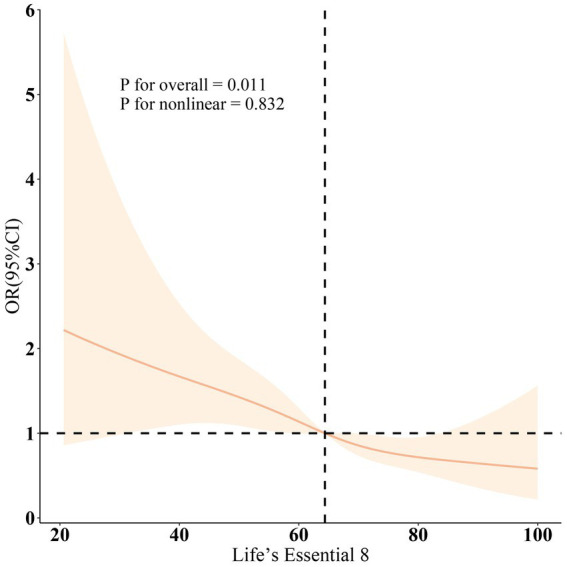
Dose-response relationships between LE8 and PD. OR (solid lines) and 95% confidence levels (shaded areas) were adjusted for age, gender, education level, marital, PIR, race, smoking, drinking, hypertension, diabetes, and high cholesterol.

**Figure 3:**
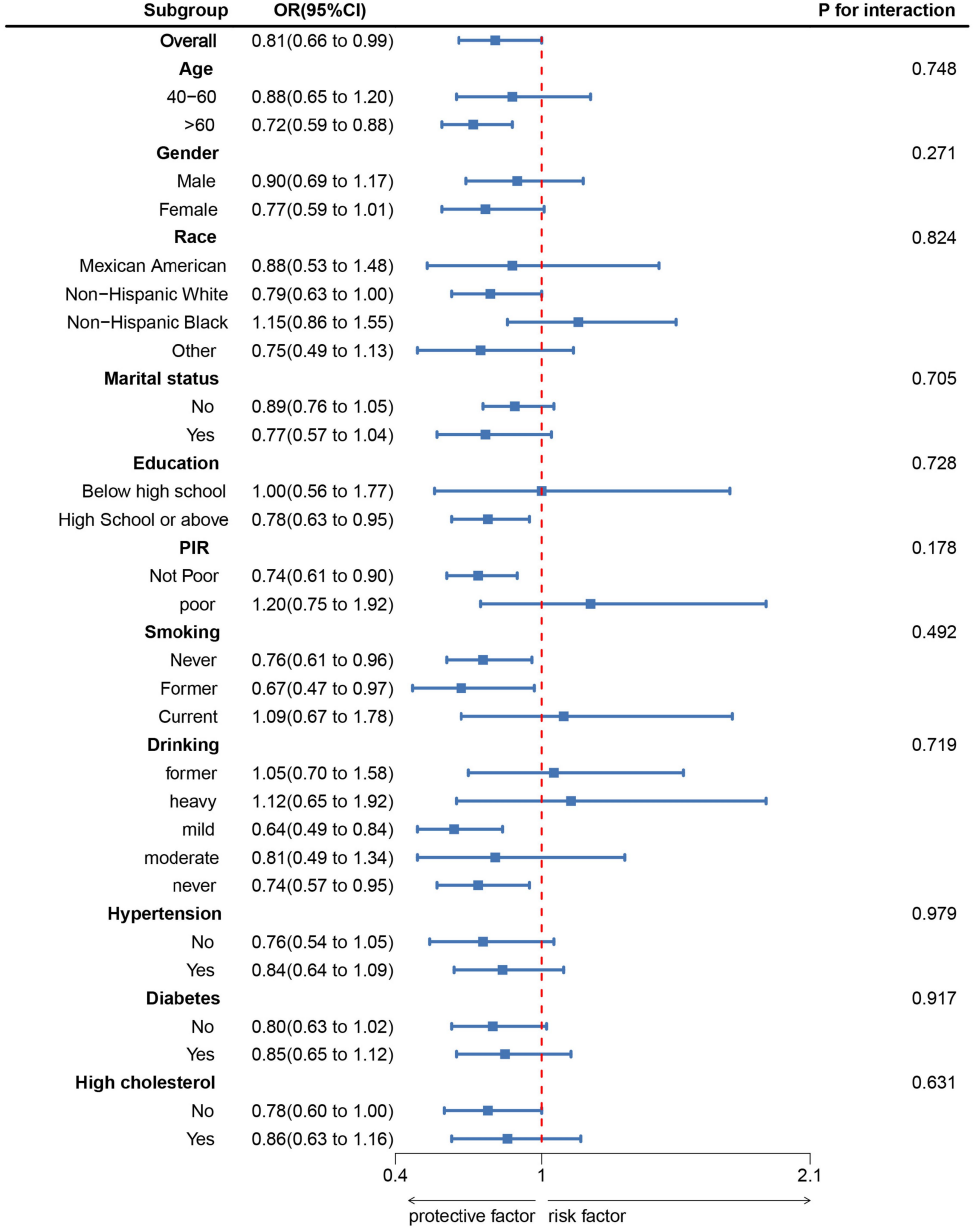
Subgroup analysis between LE8 and PD. ORs were calculated as per 10 scores increase in LE8. Analyses were adjusted for age, gender, education level, marital, PIR, race, smoking, drinking, hypertension, diabetes, and high cholesterol.

Figure 2 corroborated that LE8 exhibited an inverse association with incident PD as indicated by the RCS results. Subgroup analyses (Figure 3) demonstrated that there were no significant interactions observed between LE8 and the stratification variables, which included age, sex, race, marital status, education, economic status, smoking, drinking, hypertension, diabetes, and high cholesterol (*p* > 0.05). This relationship was found to be stable across the various subgroups analyzed.

**Figure 4:**
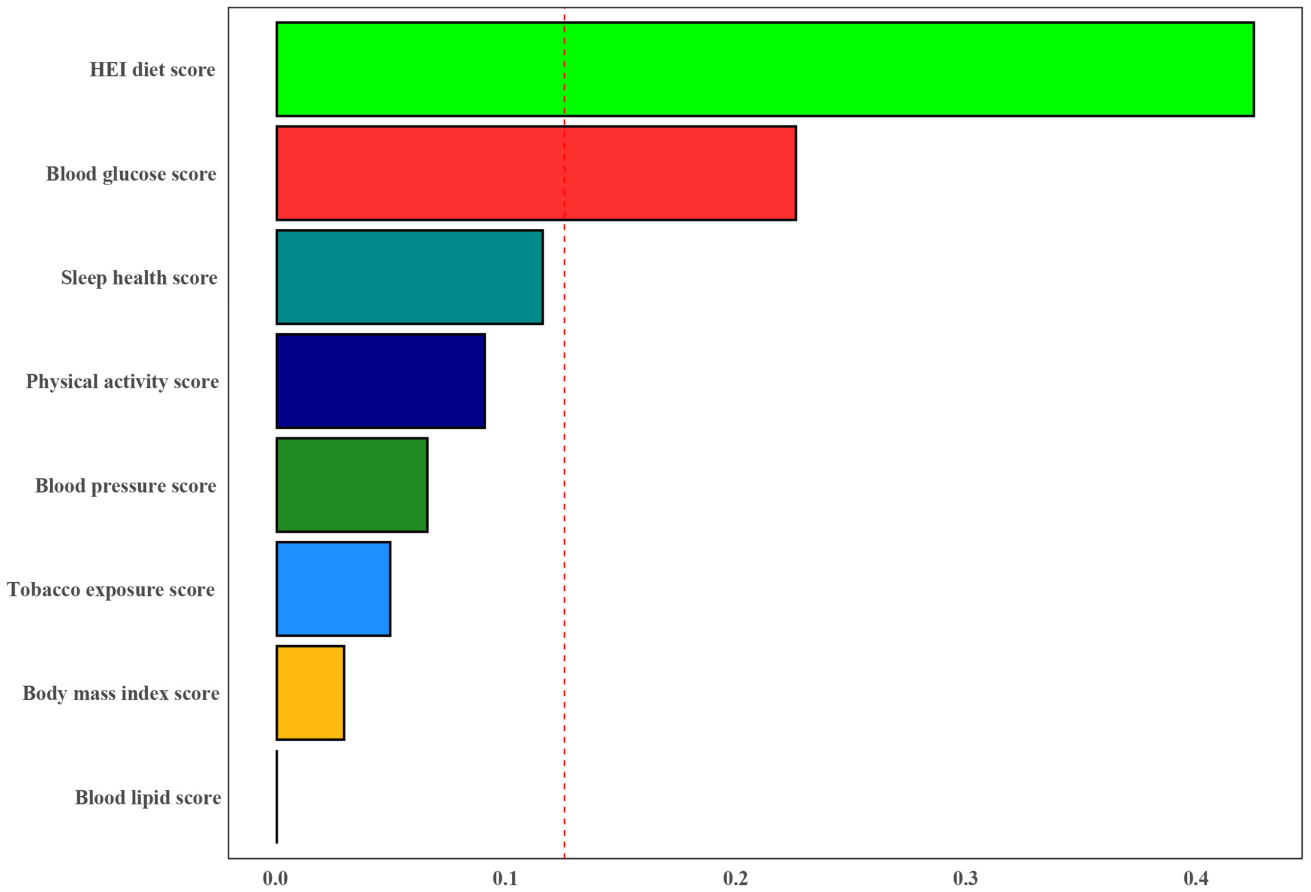
Weights represent the proportion of partial effect for each LE8 metric in the WQS regression. Model adjusted for age, gender, education level, marital, PIR, and race.

Furthermore, the WQS index derived from WQS regression demonstrated a negative association with the risk of PD (OR 0.60, 95% CI 0.40 to 0.90) (Supplementary Table S4). Figure 4 illustrated that all LE8 subgroups examined exhibited negative associations with PD, with dietary metric (weight = 0.424) identified as the most influential factor affecting the presence of PD, followed by blood glucose (weights = 0.225).

## 4 Discussion

This study examined the association between LE8 and PD using NHANES data. The findings revealed a negative association between the LE8 and the prevalence of PD. Furthermore, a dose–response relationship showed that LE8 was linearly related to PD. In the subgroup analyses, the results remained consistent and robust. In addition, WQS analyses showed that among the eight LE8 indicators, dietary factors and glycaemic health were identified as the main factors for the negative association between LE8 and PD.

This study represents the first investigation into the association between the new cardiovascular health (CVH) metric, Life’s Essential 8 (LE8), and the prevalence of Parkinson’s disease (PD). Previous research has primarily focused on the relationships between individual components of LE8 and PD. For instance, studies have suggested that poor dietary patterns may increase PD risk (Alcalay et al., 2012), while moderate physical activity may reduce it (Fang et al., 2018). Furthermore, smoking has been associated with a lower PD risk, although the mechanisms underlying this association remain unclear (Breckenridge et al., 2016). Regarding blood pressure, a meta-analysis indicated that hypertension might increase PD risk (Chen et al., 2019). Poor glycemic control has also been linked to an elevated risk of PD (Rhee et al., 2020). In terms of cholesterol, some studies have found that higher cholesterol levels may serve as a protective factor against PD (Huang et al., 2011). Lastly, sleep disorders are considered one of the early symptoms of PD and may play a crucial role in disease progression (Postuma et al., 2013). However, these studies have considered each factor in isolation. The present research provides a comprehensive perspective on the potential association between cardiovascular health and PD by evaluating the composite LE8 score.

Cardiovascular health plays a significant role in the pathogenesis of Parkinson’s disease (PD). Evidence suggests that poor cardiovascular health, including hypertension, atherosclerosis, and chronic cardiovascular metabolic diseases, is closely linked to neuroinflammation, oxidative stress, and cerebrovascular dysfunction in the central nervous system (Gorelick et al., 2011). These mechanisms may accelerate α-synuclein deposition in the brain and impair the survival of dopaminergic neurons, thereby increasing the risk of PD (Olanow and Tatton, 1999). Additionally, the health behaviors included in Life’s Essential 8 (such as a healthy diet, physical activity, and optimal blood glucose levels) are associated with reduced cardiovascular risk and may also have neuroprotective effects through improving systemic metabolic status and reducing neuroinflammation. For example, studies have shown that a healthy diet (such as the Mediterranean diet) is linked to a lower risk of PD, potentially due to its anti-inflammatory and antioxidant properties (Alcalay et al., 2012). Similarly, regular aerobic exercise has been shown to improve brain blood flow and enhance neurotrophic factors, which may reduce the risk of PD (Ahlskog, 2011). In our study, we found that individuals with higher LE8 scores had a lower prevalence of PD, which aligns with the hypothesis that those with better cardiovascular health may have a reduced risk of neurodegenerative diseases.

In this study, we used weighted quantile sum (WQS) regression analysis to identify diet and blood glucose as key factors influencing the development of Parkinson’s disease (PD) in the context of the LE8 score. This finding further underscores the critical role of dietary habits and glycemic health in PD prevention. First, regarding the relationship between diet and PD, previous research has shown that healthy dietary patterns, such as the Mediterranean diet, which is rich in antioxidants, can reduce PD risk by mitigating oxidative stress, lowering inflammation, and improving mitochondrial function (Feng et al., 2020). Consistently, our WQS regression analysis identified dietary health scores as a significant factor influencing PD risk, suggesting that healthy eating habits may play an active role in neuroprotection. Specifically, foods rich in antioxidants, such as fruits, vegetables, and whole grains, help alleviate oxidative stress, a key mechanism in PD pathogenesis, especially in the context of dopaminergic neuron damage (Alcalay et al., 2012). This finding supports the potential benefits of a healthy diet in preventing neurodegenerative diseases. Second, diabetes and hyperglycemia are recognized as risk factors for PD (Rhee et al., 2020). Additionally, studies have found that even mild insulin resistance, in non-diabetic individuals, may be associated with early PD symptoms, such as olfactory impairment, further suggesting the potential role of abnormal glucose metabolism in PD (Cullinane et al., 2022). Mechanistically, the interaction between glucose metabolism abnormalities and PD onset is likely multifactorial. Insulin not only regulates peripheral glucose metabolism but also plays a vital role in the central nervous system. Insulin receptors are widely distributed in brain regions such as the hippocampus, cortex, and substantia nigra, where insulin participates in processes like neuronal survival, synaptic plasticity, and energy metabolism. In type 2 diabetes (T2DM), insulin resistance diminishes the peripheral tissue response to insulin and impairs central nervous system insulin signaling. This impaired signaling may compromise dopaminergic neuronal function, contributing to neurodegeneration, a hallmark of PD pathology. Hence, insulin resistance is considered a key mechanism linking metabolic dysfunction and PD. In a hyperglycemic state, increased reactive oxygen species (ROS) exacerbate oxidative stress, leading to further damage to dopaminergic neurons. Additionally, insulin resistance and hyperglycemia may trigger both systemic and central nervous system inflammation, accelerating neurodegenerative processes. Mitochondrial dysfunction also plays a significant role in hyperglycemia-related mechanisms, as damaged mitochondria fail to provide sufficient energy for neurons, accelerating neuronal death (Cullinane et al., 2022). Recent studies have suggested that drugs improving insulin resistance may hold great potential for PD treatment (Standaert, 2024; Nowell et al., 2023). Our study, utilizing the large-scale NHANES database, further confirms the critical role of blood glucose factors in PD development.

It is worth noting that previous research has observed an inverse association between smoking and PD risk, known as the “smoking paradox,” where smokers have a lower risk of developing PD (Ben-Shlomo et al., 2024). However, despite the unclear mechanisms behind this phenomenon, the adverse health effects of smoking are undeniable, particularly regarding cardiovascular and lung health. Thus, smoking cessation remains an essential component of the LE8 health score. While the relationship between smoking and PD may be complex, smoking cessation is undeniably important for overall health maintenance.

This study has several notable strengths. Firstly, we utilized the extensive National Health and Nutrition Examination Survey (NHANES) database, which boasts a large sample size and national representativeness. This significantly enhances the external validity and generalizability of the study findings. The diversity and comprehensiveness of NHANES data allowed us to explore the potential associations between LE8 and PD in-depth, providing valuable insights into this field. Secondly, we rigorously adjusted for multiple potential confounding factors, including age, gender, race, education level, socioeconomic status, lifestyle habits, and physical condition. This significantly improved the reliability and accuracy of the study results. By controlling for these potential influences, we were able to more precisely evaluate the independent association between LE8 and PD. Additionally, we conducted detailed subgroup analyses to examine the relationship between LE8 and PD across different demographic and clinical characteristics. This not only added depth to the study but also provided important evidence for personalized prevention and intervention strategies. This multi-layered analytical approach enabled us to gain a more comprehensive understanding of the potential role of LE8 in PD development, offering valuable directions for future research and clinical practice.

Moreover, this study has several notable limitations. (1) Due to its cross-sectional design, we cannot establish a causal relationship between LE8 and PD; we can only infer their correlation. This design does not reveal whether changes in LE8 metrics lead to altered PD risk or whether PD itself affects patients’ LE8 scores. (2) Although we accounted for multiple known confounding factors in our analysis, we cannot exclude the influence of all potential confounding variables. For example, certain unmeasured genetic factors or environmental exposures might simultaneously affect LE8 scores and PD risk. Additionally, the NHANES database itself has inherent limitations. For instance, the diagnosis of PD primarily relies on self-reports or medical records, which might lead to some misdiagnoses or missed diagnoses. (3) Due to the cross-sectional nature of NHANES data, we cannot assess the impact of changes in LE8 metrics over time on PD risk, which could be an important aspect of understanding the relationship between the two. (4) This study relied on self-reported use of anti-PD medications to identify PD cases, which may carry a risk of misclassification or underreporting. Self-reported data can be subject to recall bias or incomplete information, potentially failing to accurately identify some PD cases. Furthermore, the lack of neurologist-confirmed clinical diagnoses or other objective biomarkers reduces the precision of the PD case identification method and may affect the validity and reliability of the findings. While the NHANES dataset offers a large and representative sample, this inherent limitation of self-reported diagnoses should be considered when interpreting the results. Future studies could strengthen these findings by incorporating clinical diagnoses by neurologists, neuroimaging evidence, or biomarkers to reduce information bias and enhance scientific rigor. (5) In this study, we utilized data from NHANES 2005–2018, with fasting glucose and glycated hemoglobin (HbA1c) used to define diabetes. However, we acknowledge that the glucose measurement methods were updated during the 2015–2018 cycle, which could result in minor variations in the reported values. While we did not adjust for these changes using forward or backward calibration equations in this analysis, future research should consider these approaches to minimize potential bias caused by methodological discrepancies. Furthermore, although we updated our analysis to use fasting weights, combining data across different cycles may still introduce some unavoidable systematic biases. (6) One limitation of our study is the absence of population-attributable fraction (PAF) analysis, which could have provided additional insight into the proportion of Parkinson’s disease cases that might be preventable by optimizing Life’s Essential 8 (LE8). Future studies need to include PAF analysis to enhance the applicability of findings to public health strategies. (7) A limitation of this study is the use of weighted quantile sum (WQS) regression, which may not fully account for the complexities of complex survey designs, including stratification, clustering, and unequal weighting. These factors could impact the interpretation and generalizability of the findings. Future studies may benefit from alternative statistical methods that can better handle such complexities and provide more accurate results.

## 5 Conclusion

In conclusion, our research findings indicate an inverse correlation between LE8 and Parkinson’s disease (PD), suggesting that improving lifestyle and health behaviors, particularly optimizing dietary habits and controlling blood sugar levels, may help reduce the risk of PD. Healthcare professionals should incorporate LE8 into patient education and prevention strategies, encouraging patients to adopt a healthy lifestyle. This approach could not only potentially lower the risk of PD but also enhance overall health and improve quality of life.

## Data Availability

The datasets presented in this study can be found in online repositories. The names of the repositories and accession number(s) can be found below: Publicly available datasets were analyzed in this study. This data can be found at: https://www.cdc.gov/nchs/nhanes/.

## Footnotes

^1^ https://www.cdc.gov/nchs/nhanes/index.htm
